# Implementing community participation through legislative reform: a study of the policy framework for community participation in the Western Cape province of South Africa

**DOI:** 10.1186/1472-698X-12-15

**Published:** 2012-08-25

**Authors:** Benjamin Mason Meier, Caitlin Pardue, Leslie London

**Affiliations:** 1 Global Health Policy, Department of Public Policy, University of North Carolina at Chapel Hill, 218 Abernethy Hall, CB #3435, Chapel Hill, NC, 27599, USA; 2 Behavioral Health Policy Associate, Voices for Children in Nebraska, 7521 Main Street, Suite 103, Omaha, NE, 68127, USA; 3 Head of the Health and Human Rights Division, School of Public Health and Family Medicine, Faculty of Health Sciences, University of Cape Town, Observatory 7925, Cape Town, South Africa

**Keywords:** Participation, Rights-based policy, Community health committees, South Africa

## Abstract

**Background:**

Amidst an evolving post-apartheid policy framework for health, policymakers have sought to institutionalize community participation in Primary Health Care, recognizing participation as integral to realizing South Africa’s constitutional commitment to the right to health. With evolving South African legislation supporting community involvement in the health system, early policy developments focused on Community Health Committees (HCs) as the principal institutions of community participation. Formally recognized in the National Health Act of 2003, the National Health Act deferred to provincial governments in establishing the specific roles and functions of HCs. As a result, stakeholders developed a Draft Policy Framework for Community Participation in Health (Draft Policy) to formalize participatory institutions in the Western Cape province.

**Methods:**

With the Draft Policy as a frame of analysis, the researchers conducted documentary policy analysis and semi-structured interviews on the evolution of South African community participation policy. Moving beyond the specific and unique circumstances of the Western Cape, this study analyzes generalizable themes for rights-based community participation in the health system.

**Results:**

Framing institutions for the establishment, appointment, and functioning of community participation, the Draft Policy proposed a formal network of communication – from local HCs to the health system. However, this participation structure has struggled to establish itself and function effectively as a result of limitations in community representation, administrative support, capacity building, and policy commitment. Without legislative support for community participation, the enactment of superseding legislation is likely to bring an end to HC structures in the Western Cape.

**Conclusions:**

Attempts to realize community participation have not adequately addressed the underlying factors crucial to promoting effective participation, with policy reforms necessary: to codify clearly defined roles and functions of community representation; to outline how communities engage with government through effective and accountable channels for participation; and to ensure extensive training and capacity building of community representatives. Given the public health importance of structured and effective policies for community participation, and the normative importance of participation in realizing a rights-based approach to health, this analysis informs researchers on the challenges to institutionalizing participation in health systems policy and provides practitioners with a research base to frame future policy reforms.

## Background

Community participation is crucial to realizing a rights-based approach to health, yet many health systems have not enacted policies to enable the institutions necessary for effective participation. With participation both a human right in itself and instrumental to the realization of other rights, this study investigates the challenges to developing health policy for community participation in the Western Cape province of South Africa. Although the Western Cape has taken evolving steps to institutionalize participatory frameworks through community health committees (HCs), these committees remain informal under the District Health System and have struggled to promote community participation without formal recognition under law. In an effort to overcome these challenges, stakeholders have sought to formalize the structures of participatory institutions through the development of a Draft Policy Framework for Community Participation in Health (Draft Policy). This experience of developing policy to meet the needs of the community and increase community participation presents an insightful case study of policy reform to institutionalize participation, addressing the complex realities of the participation process and highlighting limitations in the development and implementation of rights-based health policy.

This article examines the political, historical, and legal context for the development and implementation of the Draft Policy, analyzing key facilitating and inhibiting factors for community participation. In addressing the policy landscape in which these reforms were developed, this article reviews evolving policy efforts to provide for community participation in health systems, examining the prospective benefits of participation in local health systems and the policy efforts advanced in South Africa to ensure these benefits within a health system undergoing enormous post-apartheid transformation. With the Draft Policy as a frame of analysis, the researchers conducted documentary policy analysis and semi-structured interviews with key informants on the development of this participation policy. Moving beyond the specific and unique circumstances of the Western Cape, this analysis seeks to map the paths through which community participation is structured, functions, and relates to other sectors of society and to develop more generalizable factors for future research on community participation policy in the health system. By focusing on those thematic factors likely to facilitate or inhibit health reform, analyzing the relationship between policy frameworks and community participation, this study outlines best practices in developing policy to realize meaningful community participation in the health system and advance the progressive realization of the right to health.

### Community participation in health systems

Community participation in the health system has come to be seen as a key component of any rights-based health policy. An instrumental aspect of the human right to health—and an independent human right, an end unto itself—such participation allows for sustainable health services that more effectively address local needs [1]. With international consensus that participation is a principal component of Primary Health Care, a rights-based accord established in the Declaration of Alma-Ata [2], advocates have sought to translate this international health and human rights consensus into domestic policy. If mechanisms for community participation within the health system are structured through the development of appropriate institutions, it is thought that such participation has the potential to increase awareness of community-specific health issues, disseminate knowledge and health education, and increase accountability for health – with participation policy necessary to realize a rights-based approach to health [3,4].

Operating at an individual level, participation leads to an increased sense of partnership, decreasing isolation by fostering improved relationships between health providers and the community, and thereby positively affecting individual beliefs about government [5]. With active public participation, individuals become a part of collective efforts to assess health needs, collaborate with others, and evaluate the reform of health care programs [6,7]. Further, when communication occurs between the health system and individuals, these individual community members may be more inclined to learn about health issues specific to their community, with education influencing lifestyle choices and overall wellbeing [8-10]. This beneficial individual involvement is highly dependent on building positive relationships, two-way interactions, and effective communications within the community and across health care providers. When the relationship between health providers and community members is based on mutual respect and trust, the dynamic changes from a submissive patient passively receiving care to an informed citizen who is actively engaged in a community partnership, improving health outcomes.

At the community level, such participation facilitates greater policy responsiveness to community needs [11], contributing to the system’s effectiveness and sustainability by providing feedback and securing involvement in collective decision making [12]. When communities are well informed on health issues, their active participation in a transparent system can serve to hold service providers and government officials accountable for their actions [13]. Creating a public sphere for dialogue in the health system, such collective deliberation is seen to improve both community development and health system management, resulting in more reasoned, informed, and public-oriented decisions [14]. Through this public deliberation, community members can create avenues to exert influence on policy makers, holding providers accountable for ensuring that funds are allocated equitably and efficiently and assuring that health practitioners and community members continue to interact in respectful and constructive ways [8,15]. As a result of this participatory priority setting, the health system becomes more tailored to the specific community’s health needs and thus more likely to improve health for all.

Providing democratic legitimacy and rights-based accountability to the health system, community participation has recently become a focus of civil society and a lynchpin of health governance; yet, despite widespread theoretical consensus on the benefits of community participation in the health system, there remains little understanding of the policies necessary to facilitate community involvement. In structuring this participation, it is thought that participatory structures must be institutionalized under law in order to safeguard a political space where communities are able to form their own identity and community voice [16]. Without this deliberate and established space for community participation, such participation risks becoming a fleeting and fragmented practice, often dependent on a few charismatic and determined individuals, rather than a common practice, a political norm, and a rights-based obligation [17,18]. When policies are codified, participation can move away from being controlled by a few powerful individuals, providing a right of participation to the marginalized and, in turn, ensuring sustainable representation [19]. While studies of community participation structures in the health system have been undertaken in the United States [20], Canada [21], and Great Britain [22], there is comparatively far less understanding of how such policies have developed outside the West, especially in Sub-Saharan Africa [23]. In this process of translating international rights into national policy, creating a locally-relevant definition of health-related rights, South Africa provides a paradigmatic case study of the struggles in creating structures to assure the benefits of community participation.

### South african policy reforms to realize participation

**Figure 1:**
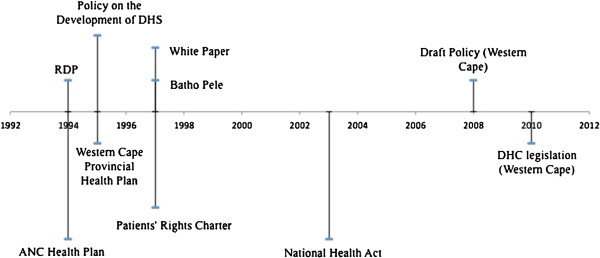
Evolving South African Reforms to Realize Community Participation.

South Africa has developed evolving executive and legislative measures to realize the benefits of community participation, establishing HCs as participation structures to promote community involvement in the health system, create sites for health-related rights, and reflect fundamental values of the New South Africa [24,25]. Through these evolving reforms, as diagrammed in the timeline in Figure 1 below, national policy has emphasized community participation as a means to assure health for all. 

With roots in the anti-apartheid struggle and the Mass Democratic Movement’s engagement with the health sector, the struggle for freedom became intertwined with the assertion of a human right to health [26]. In the presence of highly discriminatory governmental health services during the apartheid era, civil society organized to develop health services independent of the government [27]. Through the development of HCs, civil society representatives monitored health in their respective regions and acted as liaisons between the community and independent health institutions, advocating for improved services as a part of national advocacy to facilitate equity in the health system. In the aftermath of the apartheid regime, the new democratic government under the African National Congress (ANC) strove to include community participation at various levels of policy making [28], seeking to bring together “municipal authorities, unions, civics, parties, business groups, and other stakeholders into loosely corporatist bargaining structures” [29]. Applied to the health system, efforts to develop structures for community participation took shape as early as the implementation of the 1994 Reconstruction and Development Programme (RDP).

Bridging the deep socioeconomic divide left in the wake of apartheid policies, the RDP recommended fundamentally changing the South African health system to a District Health System (DHS) based on the principles of Primary Health Care, emphasizing participatory systems for comprehensive care in accordance with international consensus codified in the Declaration of Alma-Ata [30]. As a reflection of the ANC Health Plan, policymakers intended this new DHS to allow greater local control over health policy, explicitly recognizing an emphasis on “*community participation and empowerment*, intersectoral collaboration and cost-effective care, as well as an integration of preventive, promotive, curative and rehabilitation services” [31] (emphasis added). Yet despite these efforts to address health inequity through a post-apartheid system based on decentralization and community participation [8], programmatic efforts to foster a “people-driven” movement were not operationalized, as practical avenues for community participation were significantly limited because of undefined responsibilities and inadequate infrastructures [32,33].

Clarifying and extending the RDP in the development of the health system, South African policy frameworks continued to emphasize community participation in improving communication between government systems and community members. The 1997 White Paper on Transformation of the Health System in South Africa (White Paper) elevated South Africa’s continuing commitment to community participation, seeking “to foster community participation across the health sector” and establishing practical mechanisms to improve communication between the community and health services system [34]. That same year, the 1997 Public Sector Code (referred to as the Batho Pele (“people first”) Principles) proclaimed that access to public services is a right of all citizens and that communities should participate in the planning of such services to improve and optimize service delivery. To make explicit the elements of the White Paper and Batho Pele, framing government responsibilities for open communication between health service providers and citizens, the 1997 Patients’ Rights Charter (Charter) created a common standard of rights to assure that all people have access to health care. In proclaiming the rights of patients, the Charter provides standards by which patients should actively participate in the health system and play a central role in decisions regarding their own health and wellness [35]. These rights and responsibilities for individual participation would contribute to shaping the implementation of community participation through the DHS.

### Participation through the District Health System

The development of a decentralized DHS as the basis for a transformed post-apartheid health service has been at the core of policy reforms to bring together the multitude of disaggregated apartheid-era health systems and, consistent with the Primary Health Care approach, “ensure that emphasis is placed on health and not just on medical care” [36]. Facilitating participation by focusing health care decision making at the district (rather than national) level, the DHS is intended to increase communication between providers and citizens, and give communities greater opportunity to contribute to policy decisions, with the 1995 Policy for the Development of the District Health System outlining that:

"The users of these facilities should be an integral part of the health services, and not merely be seen as the passive recipients of services. In order for this to happen, the users need to be organized into a structure which will relate to the health system, and it is suggested that the structure be the *Community Health Committee*[37] (emphasis added). "

With structures for community participation central to the future implementation of the DHS, participation was seen as a means for Primary Health Care to be delivered in a more equitable and efficient way across a limited geographic district [38], bringing together all the organizations and individuals in the health system—whether governmental, non-governmental, private, or traditional—and securing the partnerships of individuals, communities, and health care providers necessary to improve health.

**Figure 2:**
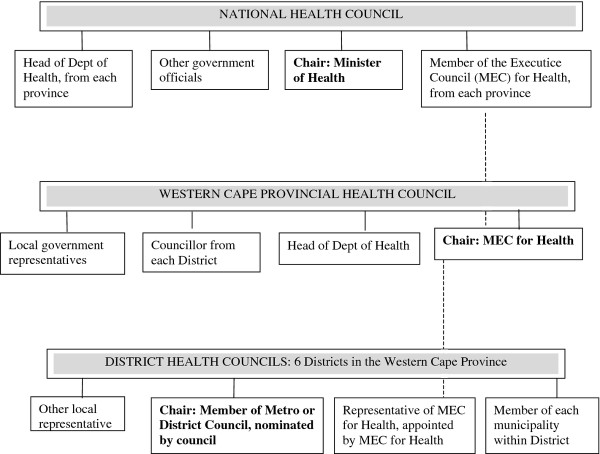
National Health Act Structures under District Health Councils.

Developed over almost a decade of negotiation among competing constituencies, the National Health Act of 2003 consolidates many of the earlier health policy developments on the DHS and, among its restructuring provisions, outlines in broad terms the governance roles of District Health Councils and the structures of HCs [39]. Figure 2 outlines the institutional landscape of these District Health Councils and the governance functions of organizations and individuals as described in the National Health Act. 

However, while the National Health Act describes both the establishment of District Health Councils (section 31) and the incorporation of HCs in every health facility (section 41), it does not explicitly link these two institutions or structure the relations between them [25], delegating authority to provincial governments to provide legislation for the functioning and management of HCs. Notwithstanding this specific mandate for the establishment of HCs under provincial legislation, the National Health Act has not been implemented to establish HCs in most of the nine provinces [25]. Although the national government has acknowledged the importance of participation in the health system, this commitment has not translated into tangible policy results at the provincial level – with a 2003 survey finding that HCs were established in only 60% of Primary Health Care facilities in the country [40] and a 2006 study concluding that many established HCs were ineffective and poorly functioning, with HC members reporting that their opinions were neither valued nor considered within the health system [41].

### The DHS in South Africa’s Western Cape

In South Africa’s Western Cape, policy makers began outlining provincial frameworks for community participation after the enactment of the 2003 National Health Act, with the roles and responsibilities of HCs formally proposed for the first time in the 2008 Draft Policy. Reflecting the structures of the National Health Act, the Draft Policy defines and describes how each clinic in the province develop an HC—composed of community members, the ward councilor, and the health facility manager—with the HC acting as a liaison between the clinic, the community, and the government and thereby facilitating conflict resolution through community representation. As a means of local conflict resolution, complaints and concerns within the community are to be addressed by the clinic’s HC, where such concerns would be discussed with clinic staff and resolved directly at the clinic level. In community representation, HCs are expected to meet with health managers (government officials and hospital representatives) in each sub-district to discuss common issues and successful strategies for health promotion. Translated into health policy, HC representatives from each sub-district then meet together as the Cape Metro Health Forum (CMHF), which further engages with civil society and government officials in policy reform efforts. This tiered system of resolution and representation envisaged in the Draft Policy provides institutional avenues to make the benefits of community participation a reality in the Western Cape.

**Figure 3:**
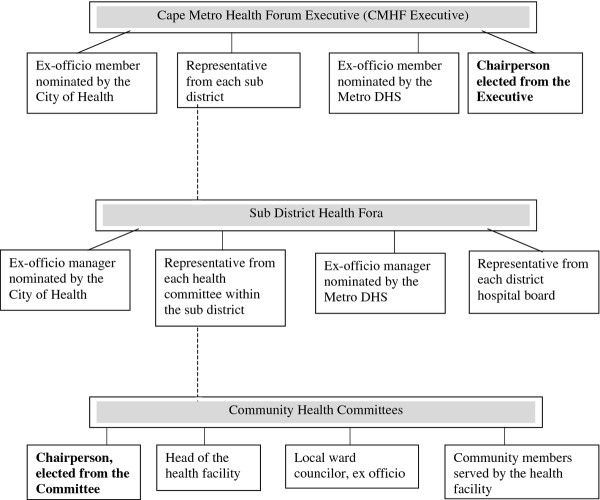
Western Cape Provincial Health Plan Structures for Community Participation in the Western Cape Province.

Established in 1995 under the Western Cape Provincial Health Plan, the CMHF forms the current structure for community participation within the Western Cape – with members and functions designated, as detailed in Figure 3, under the Cape Metro Health Forum Executive (CMHF Executive), the Sub-District Health Fora, and the Community Health Committees (HCs). While not originally intended to serve as a basis for HC interaction—with the CMHF predating the National Health Act and serving initially to provide a forum for civil society to engage with the state—the CMHF evolved to reflect its current structure for community participation.

 • Structured within the DHS, the consolidated CMHF encompasses a single Health District (with 8 Sub-District Health Fora) and is intended to include all HCs linked to clinics in the Cape Metro area (with over 80 committees as of December 2010). Representing the entire metropolitan district, the CMHF Executive comprises an elected Chairperson, representatives from each of the 8 sub-districts, and ex-officio members nominated by government health officials, all coming together to coordinate the Sub-District Health Fora, create strategies for optimal community participation structures, and evaluate the effectiveness of the HCs.

 • At the sub-district level, where health disparities lead to sub-district-specific health priorities [42], Sub-District Health Fora serve as a platform for sharing concerns and strategies among members in the sub-district, including a representative from each HC within the sub-district, a representative from each district hospital board, *ex-officio* members nominated by the Metro DHS and City Health, and civil society representatives.

 • Closest to communities themselves, the HC’s primary role is to serve as a liaison between the community and the clinic staff, with committee members intended simultaneously to provide governance to the clinic and to work directly with the community. Under the National Health Act’s representative structure for community participation, HCs consist of the local ward councillor, three to eight community members, and the head of the health facility – organized under a chairperson elected from within the HC.

Although these CMHF structures were originally outlined as part of the Western Cape Provincial Health Plan, the National Health Act does not include a CMHF-like structure for community participation under the DHS. To institutionalize this structure, stakeholders have called for legislative codification of the Draft Policy.

### The Western Cape’s draft policy framework for community participation

In a country with a robust rights-based tradition of community participation and specific national policies for widespread community involvement, the Draft Policy seeks to formalize community participation structures within the Western Cape DHS as a means: to legitimize the CMHF, Sub-District Health Fora, and HC participation structures within the health system; to ensure that the needs, concerns, and complaints of individuals and communities are properly addressed; and to foster community support for policies and programs. Providing a standardized framework for the establishment, appointment, and functioning of such institutions, the Draft Policy proposes a decentralized participation structure, creating a network of communication from local HCs to the CMHF and throughout the health system. By organizing participation around the existing CMHF, the ability of the health system to facilitate participation within the district is built upon a network already accepted as a legitimate structure by all actors involved in the health system. In implementing the Draft Policy, it is thought that the HCs, Sub-District Health Fora, and the CMHF would find greater authority for realizing community participation through the following principles and objectives:

Guiding principles

i) to observe the PHC [Primary Health Care] principles as articulated in the Alma-Ata declaration and the NHA [National Health Act] of 2003;

ii) to strengthen governance of service delivery structures and facilities through effective participation of civil society;

iii) to work in partnership with other stakeholders to improve the quality of care at all levels of the health system;

iv) to involve communities in health service delivery and health promotion activities;

v) to establish mechanisms to improve public accountability and promote dialogue and feedback between the public and all relevant stakeholders;

vi) to build a responsive organization within legal and political frameworks guided by the constitution and various pieces of legislation;

vii) to involve communities in various aspects of the planning and provision of health services; and

viii) to encourage communities to take greater responsibilities for their own health promotion and care [43].

Although this Draft Policy has been developed to give structure to institutions of community participation, it has not been codified in Western Cape legislation, leaving uncertainty in the CMHF’s formal role and authority for participation within the health system [44,45]. To understand the relationship between policy frameworks and community participation in health, this article seeks to elucidate the limitations in implementing the Draft Policy by documenting and analyzing the structures of community participation in the Western Cape. By focusing on those thematic factors that facilitate or inhibit health policy reform, this article highlights best practices in developing policy that realizes rights-based community participation. Moving beyond the specific and unique circumstances of the experience in the Western Cape, this analysis seeks to develop wider and more generalizable factors for future health systems research on community participation policy.

## Methods

With the Draft Policy as a frame of analysis, the researchers conducted a detailed case study analysis of the evolving policy landscape for community participation in the health system in the Western Cape province of South Africa [46]. Complementing such documentary legal and policy analysis, the researchers conducted semi-structured interviews and observed stakeholder discussions.

 • *Documentary legal and policy research–* Archival research was undertaken prior to identifying and conducting interviews with key informants in order to develop a more complete understanding of the political and social history of South Africa. Using a combination of policy research through the University of Cape Town’s library database, the National Library, and online research, the researchers reviewed documentation from meetings, reports, and charters, and created a detailed timeline of seminal policy documents in the evolution of community participation policy.

• *Semi-structured interviews* – Semi-structured interviews were conducted to clarify the unpublished political and social history of policy in the Western Cape, as well as provide a detailed account of the creation of the Draft Policy. Given the impracticability of experimental or statistical methods of analysis for assessing the process of health policy reform, a semi-structured interview methodology allowed the researchers to explore different avenues of inquiry as themes surfaced during key informant interviews [47]. Such semi-structured interviews facilitated an open-ended dialogue between interviewer and informant, providing unique data for understanding the political and social environment surrounding the development of the Draft Policy. Employing a snowball sampling method to select potential interview subjects, the researchers first identified the major participatory structures in the Western Cape health system, and from this, selected members of relevant governmental, community, and advocacy institutions during the time of the development of the Draft Policy. Contacting individuals who were directly involved in the policy development process, the researchers met with a range of stakeholders representing a wide variety of informant perspectives and experiences [48]. Selected in consultation with academics who were knowledgeable about Western Cape health policy, twelve potential informants were identified, of which eight were interviewed. These semi-structured interviews focused on the respective roles of informants in the creation of the Draft Policy, community participation debates that surfaced during the drafting process, obstacles to policy implementation and strategies employed to overcome these obstacles, and expected changes in community participation and public health. Employing a topic guide for the interviews, a single interviewer pursued key questions and topics with each informant, with the full research team continually revising and adapting the topic guide as themes emerged, allowing for the iterative development of more detailed questions for subsequent interviews and further analysis.

• *Group discussions and stakeholder observations* – Complementing stakeholder interviews, the interviewer attended a dialogue between government officials and community members at a forum sponsored by the South African NGO Coalition (SANGOCO), where community activists, NGO leaders, and Department of Health officials met to discuss health participation, capacity building, public health concerns, and conflict resolution. In addition, the interviewer attended several HC and CMHF Executive meetings, documenting the manner in which each meeting was held, the organization and content of the agenda, the roles of participants in policy debates, and the dialogue of participation. Where meetings were conducted in Afrikaans or Xhosa, participants translated the main subjects of debate after the meetings closed.

From transcriptions of the informant interviews and analysis of supplemental documents and observations, a narrative account of the policy making process was created. Through thematic analysis of these case study data—examining recurring topics, beliefs, and patterns [49]—the researchers identified and analyzed major themes in the policymaking process.

## Results and discussion

Developing policy that effectively implements rights-based community participation has long faced challenges in defining and addressing the complex realities of the participation process [50]. To achieve meaningful community participation that leads to progressive realization of the right to health, it is necessary to analyze the paths through which community participation is structured, functions, and relates to other sectors of society [51,52]. In the Western Cape, policymakers question whether the CMHF provides an accurate reflection of community needs, resources, and values in order to build partnerships through HCs for increased community participation in the health system. Where policy limitations may undercut community participation, reinforcing existing political and social structures and perpetuating health inequalities, these limitations must be explicitly addressed in the development of community participation policy [53].

In order for community leaders to be seen as authoritative spokespeople in the eyes of the community and the health system, providing a foundation for realizing the health benefits of participation, the method of selection, representation, and participation must be perceived as creating political legitimacy and procedural justice. For example, the benefits of meaningful participation cannot be accomplished if minority and disadvantaged groups are not accurately represented in participatory institutions or do not have substantial voice within the health system [8]. In defining the process by which representatives are elected, appointed, or assigned to HCs, the operational aspects of community participation must be understood before rights-based health outcomes are realized [1], as the form of selection of community members—by direct election from the entire community, election from specified interest groups, or appointment from local government—is crucial to the programmatic success of any participatory institution [54].

Beyond the representation process, the legitimacy of community participation requires that representative individuals possess sufficient knowledge of the health system and dedicated commitment to the participatory institutions. Yet representation creates a series of opportunity costs that many community members cannot afford (including lost pay, travel costs, and training difficulties [8]), and these costs may limit representation only to elites who may not be seen as legitimate spokespersons for the community at large. Even when those traditionally left out of the health system are able to be represented, the political and social dynamics may create an environment in which representation is not possible [19,55]. Therefore, policymakers must come to understand who is included in the definition of the community and who could potentially be excluded through the implementation of community participation policy.

Given these imperatives for—yet limitations to—the community participation structures outlined in the Draft Policy, this analysis identifies five structural obstacles to community participation in the Western Cape:

1. *There is organizational uncertainty as to what the role of the CMHF is or should be.* The CMHF lacks clearly defined authorities within the new DHS, as the CMHF’s informal consultative origins are not commensurate with the formal institutional arrangements that currently structure engagement within the health system.

2. *There is complexity in identifying, selecting, or electing those who truly represent the community*. Without established processes for determining community representation, it is often difficult to determine if representatives are participating in the best interests of the communities for whom they claim to speak, denying legitimacy to HC structures.

3. *There is little government support for building the capacity of community representatives.* The Department of Health has not instituted a structure to build HC capacity to engage with the health system.

4. *There is a lack of administrative training for HC members.* Once a committee member, there are few substantive training or administrative support structures to carry out required community representation functions.

5. *There is unclear commitment to implementing policy for community participation*. In the aftermath of developing the Draft Policy, policymakers have not sought to institutionalize community participation structures in implementing the DHS.

From this analysis, it becomes clear that provincial policy holds a crucial role in overcoming these obstacles, facilitating or inhibiting the development of representative institutions conducive to community participation.

### Organizational uncertainty

When the CMHF was formed in 1995, its establishment came about during a time of major restructuring in the Western Cape health system. At the national level, the Department of Health was seeking to bring together fourteen autonomous health authorities; at the local level, the City of Cape Town alone had twenty-seven distinct authorities providing health services. In this restructuring, the new Provincial Health Department sought to implement national policy by merging various health authorities under the mantle of one DHS. As a forum to discuss these structural reforms with affected communities, the CMHF served an essential, albeit informal, role in bringing together health officials and community members to collaborate and coordinate during the provincial implementation of the national Policy for the Development of the District Health System. With the Western Cape having since moved to formalize other institutional arrangements in the health system, the CMHF’s informal structures cannot effectively structure health system participation without legislatively defined roles and responsibilities.

Since the Western Cape has begun to put in place formal institutions for DHS oversight, the CMHF has not been able to collaborate adequately in a process in which it has no legislative standing or defined mandate within the DHS. This organizational uncertainty in its formal authority greatly inhibits the community’s ability to participate in sophisticated institutional arrangements and rigid lines of authority [8]. As noted by a key stakeholder, “provincial treasury, national treasury, national acts around finance determine how our budgets get formulated – a consultative body can’t really be involved in all of these processes.” Given these institutional limitations to community participation under informal arrangements, CMHF representatives expressed significant alienation from the health system, highlighting how a lack of defined authority has left the CMHF without any formalized basis to engage with the DHS.

To alleviate this organizational uncertainty, the Draft Policy was sought as a means to formalize the CMHF pursuant to provincial legislation. With no other institution responsible for community participation, compounded by a concern that the DHS has neglected the community engagement principles central to the National Health Act, stakeholders emphasized the continuing need for HCs, arguing that “it raises a concern of how seriously we take our very own policies as a government and how serious we are in the implementation of our policies and guidelines.” With growing concern that the continued existence of the CMHF will be threatened where it is not institutionalized under law, stakeholders lamented how the CMHF’s exclusion from the health system might significantly weaken or eliminate a role for community participation:

"If you really want people governing and people having a say, then the structure doesn’t create that. The structure creates a kind of opportunity of engagement, but it’s really dependent on the way we actually do it and the way we engage with it. "

As such, many community members fear that health reforms will not adequately allow for community participation, creating a pressing imperative for their efforts to secure implementation of the Draft Policy and thereby formalize the CMHF as a basis for engagement within the DHS.

### Community representation

Not fully addressed in the Draft Policy, there remains complexity in identifying, selecting, or electing community representatives to the HCs. As this problem was identified by a key stakeholder:

"It’s a highly politicized process. In my own opinion, it’s not necessarily the right people who come forward to represent their communities…The people who get elected in my personal estimation, are the wrong people who get elected for the wrong reasons, for the wrong things. And it’s not of their own doing or their own making, but it’s the motivation for stepping forward and being a community participator. "

Several stakeholders noted that the lack of clearly defined processes for representation creates an environment in which community representatives do not have a clear relationship to the communities for whom they claim to speak. Emblematic of the limitations to true representation, elections for HC members are frequently forgone in place of direct appointment from the committee chair. In situations in which these members are not elected or selected by standard procedures, engagement with community participation structures may serve only for personal enrichment, with community representation reinforcing existing bases of political and social capital and reflecting nothing more than personal opinion [56]. Rather than representing or understanding their communities, it is believed that several community representatives were motivated strongly by self-interest, volunteering to participate in health committees merely to gain the qualifications necessary to seek future employment and leaving the committee once employed (nominating a family member or friend as a replacement without any additional confirmation). With such processes undercutting efforts to achieve community participation, a stakeholder criticized:

"You know you’re speaking on behalf of a community of people. You have the responsibility and an obligation to that community…and often I find that we are dealing with personalities and I sometimes think, “Who are the voices behind these people and do they even understand those voices?” So how do they actually communicate those needs and don’t paper it with their own personal issues? "

These non-standard selection processes, allowing personal interests to play a role in joining committees and representing interests, can present potential obstacles to representation, denying HCs the impartiality, public spirit, and effective conflict resolution structures necessary for community participation.

### Institutional support

As health officials seek to engage with these community representatives, the government lacks a clear vision of how the DHS can institutionally support community engagement to promote meaningful participation in the health system. Reflecting on the relationship between the DHS and HCs, one Department of Health official noted:

"We’ve restructured the structure, but now we’re kind of working out the mechanics of the structure and how the DHS engages and how it works in practice. And part of that has to deal with the community and having a voice closer to management and informing processes. There hasn’t been a lot of energy into really grappling with that."

Despite serving as the main government entity responsible for the provision of health services, the provincial Department of Health has not traditionally held responsibility for building community capacity for participation, with many of these functions undertaken by civil society representatives rather than Department physicians [57]. The Department has been restructured to emphasize Primary Health Care and rights-based community participation; however, the Department leadership comes primarily from medical backgrounds, and stakeholders within the Department noted the enduring limitations of this medicalized workforce:

"The Department at the moment is doctor heavy and comes with the thinking around the way doctors operate and the medical model… The ideology is not developmental. It’s not rights-based. With that kind of culture, I don’t know if we are the right people to do it, even if we had some obligation to support them [community representatives]. "

As a result of this organizational culture, it was believed—both inside and outside the Department of Health—that many health officials continue to question whether the Department has the obligation or ability to engage in capacity building to support community representatives. Without institutional support to enable representatives, a stakeholder questioned: “Are we going to wait for health committees to somehow organically develop this capacity or do we actually invest in looking into how to increase capacity in health committees?” With community members voicing frustration with the Department of Health, these community members saw themselves not as partners with the Department but as “watch dogs” of the Department, lacking an ability to participate within the Department’s institutional structures.

Even with the Draft Policy, there remains ambiguity over how the Department might communicate effectively with its constituents, give voice to community representatives, and relate institutionally to community participation structures. As a stakeholder warned, “the whole idea of putting the structure in place was to bring the services closer to people, to have the decision-making processes closer to where the action is happening.. We are going to fall short if we just put the structure in place and we don’t actually stay true to the idea.” As stakeholders seek support for the Draft Policy, it is clear that legislation is only the first step, with institutional support programs necessary to build the capacity and trust of community representatives to participate in the health system.

### Administrative training

Beyond building capacity for community participation, HC members often noted the need for administrative training – as defined by communities themselves, but including, at a minimum, basic computer skills, administrative committee procedures, and information on prevailing health issues, DHS bureaucratic functions, and HC participation responsibilities. With this training only just begun, one of the trainers reflected on how training could impact the role of the community representatives in the health system:

"The interesting thing for me was that when we were doing this training that people’s eyes opened. “Yeah that makes sense.” And it was heartening to see that people did understand what’s happening to them, why they were getting sick. Because a lot of the training was around what makes you sick, what makes you better – understand Primary Health Care first before you can understand your role in the Primary Health Care system. People did want to know. People are smarter than people expect. They can work some things out because they are survivors. "

Confirming the findings of an earlier study conducted by The Learning Network for Health & Human Rights (Learning Network) at the University of Cape Town [45], the most common concern of HC members was the need for greater training in community representation and understanding of HC roles and responsibilities.

Yet among officials within the Department of Health, even among those who were otherwise supportive of community participation, there was criticism of training efforts and concern for achieving training goals. As reflected by a key Department stakeholder, “there was a big drive [for training]…but from my side, very little return for the investment.” With Department officials finding that community representatives lacked the professionalized conduct necessary to benefit from administrative training, Department representatives criticized attendees for failures in “respecting people’s time, contacting [Department members], keeping informed, making sure they arrive at the right time, constituting a proper meeting, taking proper meeting procedure… All of that is missing from this process.” While HC members regretted that unavoidable issues (such as access to transportation and prior commitments) had restricted training attendance, limiting the benefits of these previous trainings, these community representatives nevertheless emphasized the importance and success of these trainings.

Such opposing perspectives on the value and impact of training (between Department officials and community representatives) highlight the divergent ways in which the two groups define training success. Because the Department is a large governmental institution that is evaluated on the basis of achieving measureable targets within a limited budget, cost-benefit analysis defines its success or failure; in comparison, community members and trainers may gauge success on factors not amenable to quantifiable measures such as individual empowerment or community engagement [55]. Further, with this administrative training thought to provide a demonstrable impact only once a threshold number of representatives have been trained, the Department would need to scale-up training to see a measurable association between representative training and community participation.

### Policy commitment

As the health system is reorganized so that management can be brought closer to communities and communities can have a voice in policy, many question the lack of focus on effective community participation in health system management and lack of commitment to engaging HCs under the new DHS. Despite changes in national policy, many of the provincial stakeholders feel ‘stuck’ in the old system and are operating as if no change has taken place. Effectively shifting from a paternalistic medical model to a participation-based model requires a significantly different approach to health and healthcare that has not been addressed in provincial policy. With less attention paid to establishing effective institutions for community participation at the local level, policy reforms have not focused on building and supporting effective HCs. Since the adoption of the Patients Rights Charter and the White Paper in 1997, over a decade passed before stakeholders developed the Draft Policy to institutionalize HCs; and rather than adopting this Draft Policy, the eventual District Health Council Bill extends this lack of commitment to community participation and excludes HCs altogether. Supporting HCs through participatory policy would require investment in: how to mobilize communities to select representatives; how to ensure that HCs meet regularly; how to engage health services management; and how to coordinate communities with management at the local clinic level. Where leaders in the health system continue to neglect community voices, there is a need for effective and engaged policymakers who have a clear understanding of what kind of community participation is required and how such participation can be realized in a way that allows community representatives to become more active members in the policymaking process [1].

Indicative of this lack of policy commitment to community participation, the Department of Health promulgated new legislation in December 2010 to institutionalize a District Health Council [58]. Excluding a formal basis for community representation, the Western Cape District Health Council Bill defines the new District Health Council structure as including:

(a) a Chairperson, as a member of the metropolitan or district municipal council in the specific health district, nominated by the relevant council;

(b) a person appointed as a representative of the Provincial Minister;

(c) a member of the council of each local municipality within the health district, nominated by the members of the relevant council; and

(d) not more than five other persons, appointed by the Provincial Minister after consultation with the municipal council of the metro or district municipality.

While such a District Health Council could elicit community participation through its authority to “consult with or receive representations from any individuals, organizations, or institutions on any matter regarding health or health services” and to “ensure that appropriate and comprehensive information is disseminated to the local communities on the health services in the health district” [58], this new legislation never establishes formal structures for community participation.

Without requiring formal community representation under this new policy, stakeholders expect that the establishment of a District Health Council will lead to the dissolution of the CMHF, ending longstanding efforts in the Western Cape to foster formal community participation in the health system. As the Department of Health reviews previous community participation structures in preparation for the establishment of the new District Health Councils, government officials are contemplating the prospective loss of the CMHF, explaining that:

"This is going to be a challenge. Because we’ve legitimized these structures, because we interact with them, because we give them funding. And once a District Health Council comes into being, [the CMHF and sub district foras] have the perception they are legitimate. But they are actually not legally legitimate in terms of the structure. It’s going to call all of this into question. "

In the absence of legislative institutionalization, the CMHF and HCs have evolved over time to serve a quasi-official role for community participation in the health system, and yet their future is unclear.

Recognizing that the goals, expectations, and methods of community participation must be clearly defined and formally established to ensure a positive working relationship between the health system and community representatives, policymakers must outline specifically defined objectives, roles, and responsibilities to create mutually-accepted, effective, and legitimate institutions to represent the community’s needs. Through a policy framework for community participation, this new District Health Council can develop a transparent and interactive process by which the community’s specific roles are clearly defined, each representative is perceived as a valid representative of the community, capacity is built for engagement with the health system, and the health system is responsive to community concerns.

## Conclusion

With community participation vital to realizing South Africa’s commitment to the human right to health, it is crucial that policies address the institutions by which participation is established, formalized, and maintained within the health system. Because of shortcomings in community participation policy, many have come to undervalue the relationship between the government and the community since the hopeful beginnings of community participation in the New South Africa. While the Western Cape has taken evolving steps to institutionalize these participatory processes, with the development of the Draft Policy and most recently with the legislative adoption of District Health Councils, these transitions may signal the decline, demise, or complete reconfiguration of existing structures for community participation, leaving HCs without direction moving into the future.

From the Western Cape experience, many lessons emerge in the context of policy development for community participation in the health system. To assure institutional frameworks for community participation, this research finds that there must be clearly defined roles and functions of community representatives, codified in legislation, that specifically outline how communities engage with government through effective and accountable channels for participation. Facilitating this rights-based participation in the health system, ongoing training and policy support must be established to enable communities to communicate with officials. Without legislative authority that articulates participatory structures, community participation is likely to fall into uncertainty, inefficiency, and dissolution. There are abundant benefits of community participation, but these benefits have the potential to be lost in a health system in which community participation is exclusively dependent on power structures, political will, and informal institutions. Without further research to establish clear and precise roles for participatory institutions, paired with extensive training and capacity building for representatives, community participation will not be able to achieve its full potential in realizing health for all.

## Abbreviations

ANC: African National Congress; CMHF: Cape Metro Health Forum; CMHF Executive: Cape Metro Health Forum Executive; DHS: District Health System; Draft Policy: Draft Policy Framework for Community Participation in Health; HCs: Health Committees; Learning Network: Learning Network for Health & Human Rights; SANGOCO: South African NGO Coalition; White Paper: White Paper on Transformation of the Health System in South Africa.

## Competing interests

The authors declare that they have no competing interests.

## Authors’ contributions

BMM and LL conceptualized the study. CP carried out documentary research and semi-structured interviews. All three authors participated in the analysis of results and development of this manuscript.

## Pre-publication history

The pre-publication history for this paper can be accessed here:

http://www.biomedcentral.com/1472-698X/12/15/prepub
